# Multimodality Approach to Lymphedema Surgery Achieves and Maintains Normal Limb Volumes: A Treatment Algorithm to Optimize Outcomes

**DOI:** 10.3390/jcm11030598

**Published:** 2022-01-25

**Authors:** Peter Deptula, Anna Zhou, Victoria Posternak, Hui He, Dung Nguyen

**Affiliations:** Division of Plastic Surgery, Stanford University Medical Center, Stanford, CA 94304, USA; pdeptula@stanford.edu (P.D.); atzhou@stanford.edu (A.Z.); VPosternak@stanfordhealthcare.org (V.P.); huhe@stanfordhealthcare.org (H.H.)

**Keywords:** lymphedema, lymphangiogenesis, vascularized lymph node transfer, lymphaticovenous anastomosis, BioBridge

## Abstract

Surgical treatment of advanced lymphedema is challenging and outcomes are suboptimal. Physiologic procedures including lymphaticovenous anastomosis (LVA) and vascularized lymph node transfer (VLNT) improve lymphatic flow but cannot reverse fibrofatty tissue deposition, whereas liposuction removes fibrofatty tissue but cannot prevent disease progression. The adjunctive use of nanofibrillar collagen scaffolds (BioBridge^TM^) can promote lymphangiogenesis. We report a treatment algorithm utilizing a multimodality approach to achieve sustained normal limb volumes in patients with stage II-III lymphedema. A retrospective review of late stage II-III lymphedema patients treated with liposuction, physiologic procedures, and BioBridge^TM^ from 2016 through 2019 was conducted. Treatment outcome in the form of excess volume reduction is reported. Total of 14 patients underwent surgical treatment of late stage II and III lymphedema according to our triple therapy algorithm. Patients had a baseline median volume excess of 29% (19.8, 43.3%). The median volume excess was improved to 0.5% (−4.3, 3.8%) at 14.4 months from the first stage surgery (*p* < 0.05) and further improved to −1.0% (−3.3, 1.3%) after triple therapy with BB placement at 24.6 months. A triple therapy surgical treatment algorithm can optimize outcomes and achieve sustained normalization of limb volume in late stage II-III lymphedema. The incorporation of nanofibrillar collagen scaffold technology allows for improved and sustained volume reduction.

## 1. Introduction

The surgical management of lymphedema remains challenging and requires a multimodal approach to restoring the affected limb to its normal volume and function [1,2]. Current treatment options include debulking and physiologic procedures. Debulking procedures such as suction lipectomy address the bulky soft tissue excess. Physiologic procedures, which consist of lymphaticovenous anastomosis (LVA) and vascularized lymph node transplantation (VLNT) address the excess interstitial fluid. Neither of these established procedures aim at restoring the nonfunctional lymphatic channels throughout the length of the limb, which characterize later stage lymphedema.

To address this problem, our authors have begun to incorporate BioBridge^TM^ (BB) (Fibralign Corp, Union City, CA, USA) into our treatment of late-stage lymphedema [3,4,5,6]. This nanofibrillar collagen scaffold consists of highly aligned parallel channels that demonstrated efficacy in lymphangiogenesis in preclinical and early clinical studies [3,5]. Proposed mechanisms for its therapeutic effect include capillary action resulting in the initiation of interstitial flow, and also by serving as a scaffold for cellular migration, attachment, and alignment of endothelial cells [5,7].

Our authors have previously reported on a treatment algorithm for lymphedema using a two-fold combination of physiologic and debulking procedures [1]. While we have had success at maintaining normalized lymph volumes in our previous report, patients with more advanced stage II and III lymphedema have unpredictable results and pose a greater challenge at maintaining limb volume reduction in the long-term. We have further refined our algorithm for the treatment of late stage II and III lymphedema with the addition of BB nanofibrillar collagen scaffold technology. Here, we describe a new treatment algorithm consisting of a triple modality in the treatment of late stage II and III lymphedema. Our authors hypothesize that this triple therapy algorithm will result in effective limb volume reduction and allow for long-term maintenance.

## 2. Materials and Methods

A retrospective analysis of patients who underwent surgical management of lymphedema using our triple therapy algorithm was conducted from 2016–2019. Patients with late stage II and stage III lymphedema were considered for this study. Inclusion criteria were patients with a normal contralateral limb for comparison, compliance with therapy, and at least two years of follow-up. Limb volume in the affected limb of all patients was at least 10% greater than the contralateral unaffected limb. Patients with a history of cellulitis were included only after treatment and full resolution of symptoms. Preoperative patient information included age, lymphedema site, and lymphedema stage. The sequence of surgical treatment was recorded. Liposuction volumes as well as type of physiologic surgery was recorded.

A volumetric analysis was performed pre-operatively and post-operatively at regular intervals. Limb volume measurements were carried out using the truncated cone method [8,9,10,11]. Here, limb circumference is measured at 4 cm intervals to approximate limb volumes by the following formula: Limb Volume = πh(R^2^ + Rr + r^2^)/3. Here, let h be the height (4 cm), R the radius of the lower base, and r the radius of the upper base) [8,9,10,11].

Excess limb volume of the lymphedematous limb was calculated as a percentage compared to the unaffected contralateral limb. Subject demographic data area is summarized as averages with standard deviation. Statistical analysis was performed using Wilcoxon signed-rank test for repeated measures. These data are presented as median with interquartile range (IQR). Subgroup analysis was also performed for upper extremity, lower extremity, VLNT and VLNT + LVA. *p*-values of <0.05 were deemed statistically significant.

### Treatment Protocol

**Figure 1:**
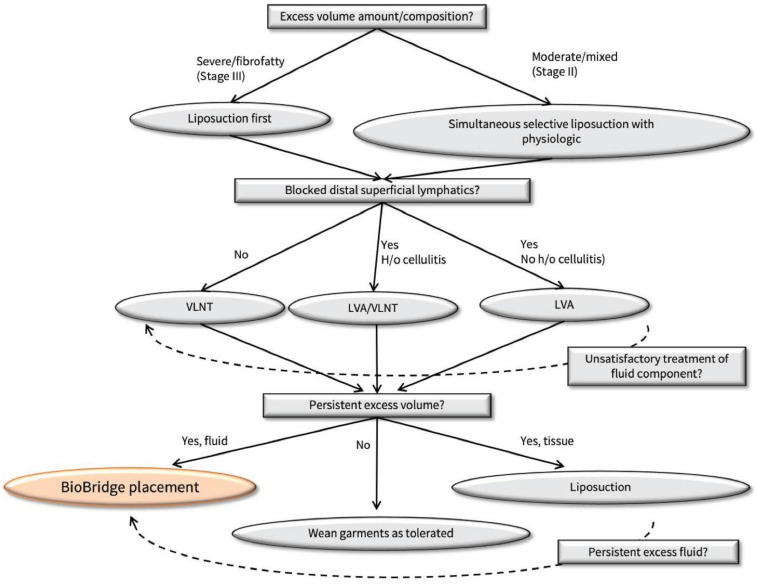
Treatment algorithm for Stage II-III lymphedema.

All patients undergoing triple therapy are first medically optimized with complete decongestive therapy including compression garments, lymphatic massage, and elevation [6,12,13,14]. Following stable medical management of lymphedema, patients were treated surgically based on our triple therapy algorithm (Figure 1). Patients with predominantly fibroadipose component of lymphedema (stage III) were treated first with liposuction debulking. After 1 year, patients then underwent lymphatic mapping to identify the presence of blocked distal superficial lymphatics. Patients who have blocked lymphatics were deemed candidates for LVA with the addition of VLNT if there was a history of cellulitis. Patients without targetable blocked lymphatics were offered VLNT.

Patients with a mixed presentation of both fibroadiposity and fluid excess (late stage II) were treated first with a combination of selective liposuction and physiologic procedure. The patients were then followed for 1–2 years post-operatively to allow for homeostasis. Any residual fibrofatty tissue is addressed with additional liposuction treatment. Lastly, excess volume in the form of fluid is addressed with BB placement to further augment lymphatic drainage. BB is placed percutaneously in the subcutaneous plane using a SecurusEP (Suture Ease Inc., San Jose, CA, USA). On average, 2–4 tracks of BB are created by tunneling the scaffold in tandem from the site of intact lymphatics distally to the LVA and/or VLNT, and then to the nearest intact nodal basin [3].

In cases where liposuction was performed alone in the first stage, dry liposuction technique was utilized and performed in a circumferential fashion [1,15]. Debulking was performed until the endpoint measure of calculated excess volume was obtained, and the limb volume approximated the unaffected contralateral limb. Postoperatively, patients were placed into Juzo class III flat-knitted compression garments custom-made to match the measurements of the contralateral normal limb, to be worn at all times except for daily changes. Patients were prescribed two sets of garments to allow for daily washing. The garments were renewed and remeasured every 3–6 months as the old garments became loose.

In cases where liposuction was performed simultaneously to physiologic procedures, a lymph-sparing technique was used [1,16,17]. Here, indocyanine green (ICG) lymphangiography and Lymphazurin (isosulfan blue) were used to identify intact lymphatic channels [18]. Wetting solution instillation was performed to select areas avoiding injury to the marked lymphatics using longitudinal, non-circumferential technique. Following physiologic procedures, the treated limb is elevated for three weeks strictly without compression. The limb is then transitioned to class I compression garment for the upper extremity and class II compression for the lower extremity at the three-week time point. After three months, all limbs are transitioned to custom class II garments made to the unaffected contralateral limb dimensions. Subsequently, garments area weaned by duration and level of compression as tolerated.

Supraclavicular, groin, and omental flaps were used as vascularized lymph node flaps. These were transferred to the axilla or antecubital fossa in the upper extremity and groin or popliteal fossa for lower extremity cases. LVA were performed in either an end-to-end or end-to-side fashion at sites of lymphatic blockage as identified by ICG lymphangiography.

## 3. Results

A total of 14 patients underwent surgical treatment of late stage II and III lymphedema according to our triple therapy algorithm. Average patient age was 62 ± 12.1 years. Total of 11 patients had late-stage II lymphedema, while three patients had stage III disease. Moreover, 8 patients had lymphedema of the upper extremity, while 6 patients had lower extremity disease. Preoperative patient data are summarized in Table 1.

Total of 11 patients with late stage II lymphedema underwent simultaneous liposuction with physiologic procedure followed by BB placement. Three patients with stage III disease underwent large volume liposuction first, followed by physiologic procedure and finally BB placement. Overall, 12 patients had VLNT performed, while 8 patients had LVA with 1–3 bypasses performed based on targetable blockages. Liposuction volumes averaged 500 ± 168 cc for the upper extremity and 1753 ± 1530 cc for the lower extremity. Surgical details of patients are summarized in Table 2.

**Figure 2:**
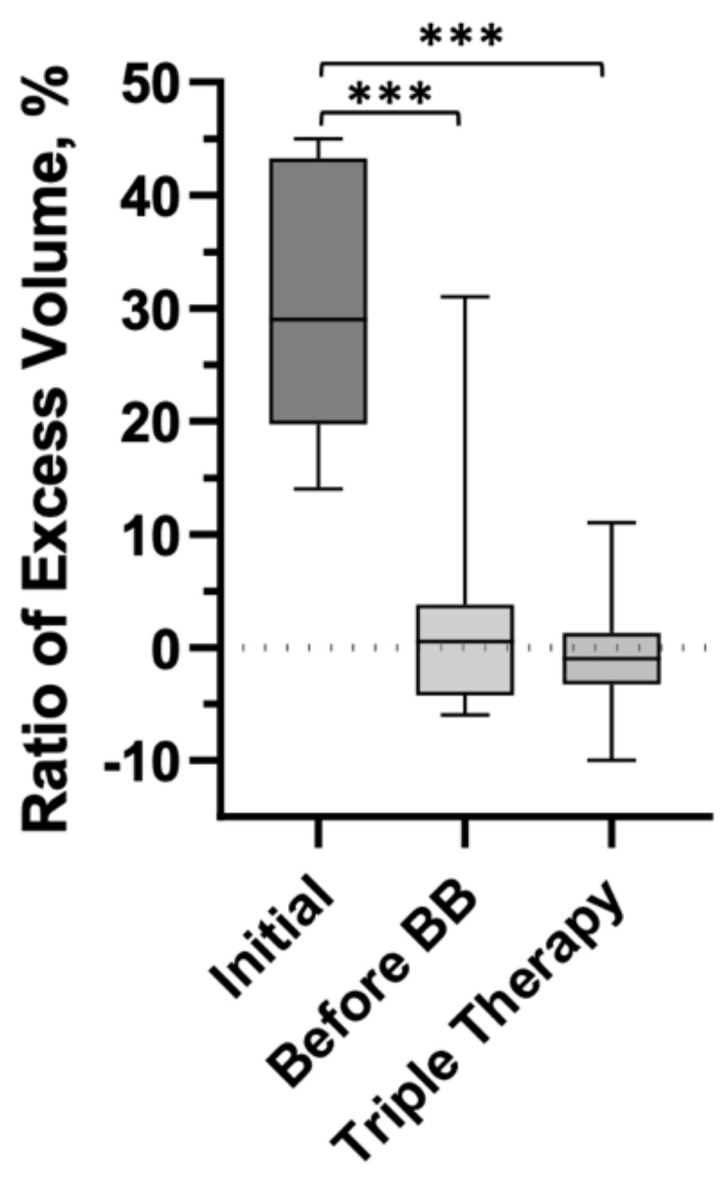
All patients combined demonstrated a median relative volume excess of 29% (19.8, 43.3%). Following liposuction and physiologic procedures, volume excess was 0.5% (−4.3, 3.8%, *p* < 0.0001). After BB placement and completion of triple therapy surgery, median volume excess was −1 (−3.3, 1.3%, *p* < 0.0001). Data are presented as median values and IQR. Signficiant values (*p* < 0.05) are indicated by (***).

**Figure 3:**
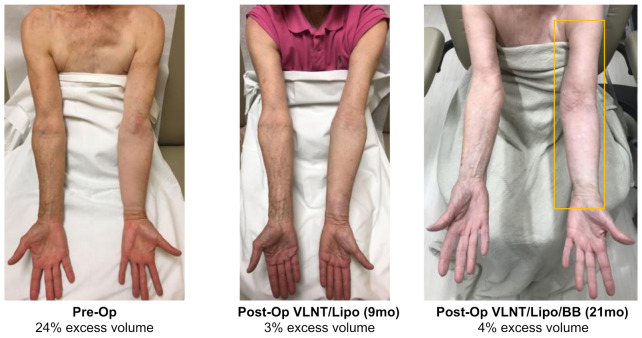
Upper extremity excess volume of 24% reduced to 3% at 9 months following combined VLNT and liposuction. BB was implanted at 12 months post-op resulting in the stabilized volume excess of 4% at 21 months following triple therapy surgical management.

**Figure 4:**
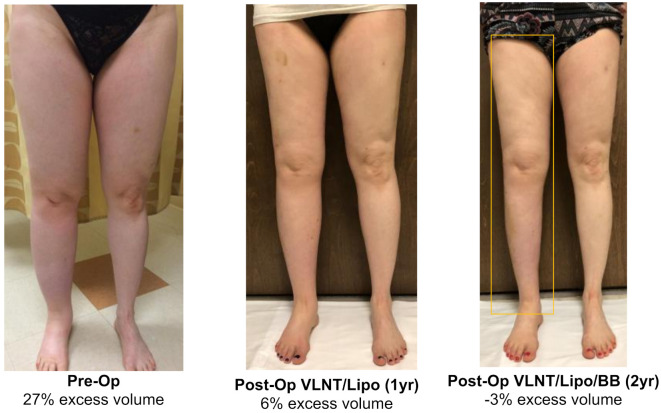
Lower extremity with 27% excess volume reduced to 6% at 12 months following VLNT and liposuction. BB placement at 1 year post-op resulted in volume excess improvement to −3% at 24 months following triple therapy surgical management.

**Figure 5:**
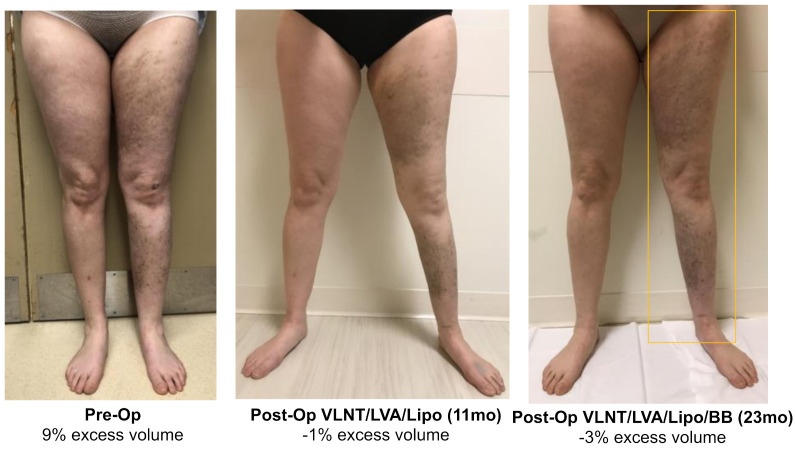
Lower extremity with 9% volume excess reduced to −1% at 11 months following VLNT/LVA and liposuction. BB placement at 12 months post-op resulted in −3% volume excess at 23 months following triple therapy surgical management.

For all patients combined, analysis demonstrated a median relative volume excess of 29.0% (19.8, 43.3%). Following liposuction and physiologic procedures, volume excess at 14.4 months postoperatively was 0.5% (−4.3, 3.8%), *p* < 0.0001. Subsequently following placement of BB, median volume excess was noted to be −1.0% (−3.3, 1.3%) at 24.6 months from the first stage surgery (*p* < 0.0001) (Figure 2). Total edema volume reduction was 95% (±28%) following liposuction and physiologic surgery. Following BB placement, total edema volume reduction was 103% (±31%). The post-operative volume reductions resulted in marked visual and symptomatic improvement (Figure 3, Figure 4 and Figure 5).

**Figure 6:**
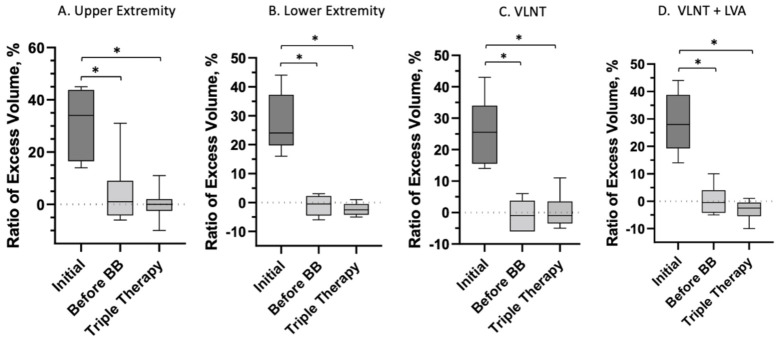
Subgroup analysis of patient median volume excess ratio at baseline, after liposuction and physiologic surgery, and after BB placement. Data are presented as median values and IQR. Statistically significant values (*p* < 0.05) are indicated by an asterisk (*).

In the 8 upper extremity patients, a median excess volume ratio of 34% (17, 44%) was observed at baseline. Excess volume improved to 1.0% (−4.3, 9%, *p* < 0.0078) after first stage surgery measured at 13 months, and 0.0% (−2.5, 2%, *p* < 0.0078) after completion of triple therapy treatment at 23 months. For the 6 lower extremity cases, median excess volume ratio of 24% (20, 37%) was identified at baseline. Excess volume ratios improved to −0.5% (−4.5, 2.3%, *p* = 0.0313) after first stage surgery at 16.7 months, and −2.5% (−4.3, −0.5%, *p* = 0.0313) after completion of triple therapy approach at 27.3 months (Table 3, Figure 6).

Physiologic surgery was performed as either VLNT, LVA, or VLNT with LVA. In the VLNT-only group consisting of 6 patients, baseline median volume excess ratio was 26% (16, 34%). Following liposuction and VLNT, median volume excess ratio improved to −1.0% (−6, 3.8%, *p* = 0.0156) at 9 months. After BB placement, median volume excess was at −1.0% (−3.5, 3.5%, *p* = 0.0313) at 19 months. In the VLNT + LVA group, baseline median volume excess was 28% (19, 39%). After liposuction and VLNT + LVA, volume excess improved to −0.5% (−4.3, 4.0%, *p* = 0.0313) at 21 months. Following BB placement, volume excess further improved to −2.5% (−5.5, −0.5%, *p* = 0.0313) at 32 months. (Table 3, Figure 6).

## 4. Discussion

Our novel triple therapy algorithm addresses each of the three pathophysiologic components of late-stage lymphedema. Liposuction addresses fibrofatty tissue infiltrate. Physiologic procedures including LVA and VLNT address excess interstitial fluid. Lastly, BB implantation addresses the nonfunctional lymphatic channels throughout the affected limb that characterize later stage lymphedema.

The predominance of fluid or fibrofatty infiltrate largely dictates the surgical approach for treating lymphedema. This distinction is determined largely by clinical exam. However, magnetic resonance has been used to refine this qualitative determination of volume excess [19]. Suction lipectomy is well-known to be effective in treating fibrofatty predominant lymphedema [15,20,21,22]. Liposuction first allows for more complete treatment area without the need to avoid the physiologic field, while also allowing for more effective post-operative compression [1]. Primary liposuction however results in greater difficulty in a second-stage physiologic procedure due to increased scar tissue [1].

A variety of techniques allow for the sparing of lymphatic channels that could be injured during circumferential liposuction [16,18,23]. These include the use of tumescent, longitudinal suction lipectomy, and avoiding preoperatively marked lymphatic channels [16,17,18,23]. A physiologic surgery first approach allows for easier dissection, and allows for accurate determination of excess fibrofatty tissue. Simultaneous liposuction and physiologic surgery allow for easy physiologic procedure site dissection, avoid disruption of physiologic surgery, and allow for fewer procedures [1]. Downsides of the combined approach are the need for lymph-sparing liposuction with avoidance of the physiologic surgery site and inability to use compression post-operatively [1].

Primary physiologic surgery is most beneficial for stage I and II disease, where volume excess is largely fluid based. These patients are likely to benefit from physiologic procedure alone and can undergo subsequent lymph-sparing liposuction should they not achieve adequate volume reduction [1]. Since the present treatment algorithm is focused on late-stage II and III disease with greater fibrofatty excess and less fluid excess, primary physiologic procedure first strategy is not employed.

An additional branch point in our treatment algorithm occurs in selection of physiologic procedure, LVA vs. VLNT. Here the presence of blocked superficial lymphatics as determined by preoperative lymphangiography determine candidacy for the LVA procedure. If no blocked lymphatic channels are identified, then VLNT is the remaining physiologic procedure of choice. Patient history of cellulitis is an indication for the addition of VLNT in our algorithm. This practice is based on previous reports of VLNT offering a therapeutic advantage over LVA in patients with a history of cellulitis [24].

Our previously reported algorithm that does not utilize BB was successful with average volume reductions of 82–106% following the dual therapy approach [1]. The current triple therapy approach yields an average excess volume reduction of 103% at 24.6 months, which represents greater volume reduction over a longer follow-up period. At just over 1 year following the combination of liposuction and physiologic surgery, a significant improvement in volume excess ratio is demonstrated in our current study (29 vs. 0.5%, *p* < 0.05). Subsequently, there is continued improvement in volume reduction (0.5 vs. −1.0%), though it is not statistically significant. However, it is the ability to help patients continue to reduce volume excess and sustain significant volume reduction out to two years postoperatively that is the highlight of our new treatment algorithm. Our authors attribute this improvement to the addition of BB in our treatment armamentarium. BB acts by facilitating lymphangiogenesis, which allow us to better address the nonfunctional lymphatic channels than we were previously [1,7]. Our subgroup analysis demonstrates that therapeutic benefit is significant in both upper and lower extremity cases of lymphedema. Furthermore, significant reduction in median volume excess ratio was significant regardless of patients undergoing VLNT only or VLNT + LVA. All subgroup analysis demonstrated significant improvement compared to baseline that was sustained out to nearly two years.

While liposuction debulking along is effective at reducing large volume lymphedema, continuous high-grade compression is required to maintain long-term results. By adding physiologic procedures to surgical treatment, we have demonstrated that volume reduction can be maintained for almost two years with less compression. Our triple therapy algorithm also pairs favorably to other studies using the combination of debulking and physiologic procedures [18,25,26]. Campisi et al. demonstrated a reduction of excess limb volume of 87–88% with LVA followed by liposuction at 5–11 months in patients with Stage II and III lymphedema [18]. Nicoli et al. reported on treating stage II lymphedema of the upper extremity with laser-assisted liposuction following VLNT [25]. Here, authors were able to achieve excess limb circumference reduction of 40–51% by 6 months [25]. Agko et al. demonstrated a 96% reduction in excess limb circumference in stage II lymphedema using double omental VLNT followed by liposuction at 6–8 months [26]. A prospective study by Di Taranto et al. also demonstrated significant improvement in limb circumference following a combined approach using VLNT, LVA, and selective liposuction [27]. While successful use of physiologic and debulking procedures is now well-described, we showed that the addition of BB into the treatment algorithm further improves lymphedema outcomes.

This study has several limitations including the retrospective nature of the study and the relatively small number of patients. As this triple therapy algorithm has demonstrated early success, we anticipate continued accrual of patients for future studies. Another limitation is our lack of a control group with randomization of treatment arms. Based on these findings, future prospective studies are underway examining the effect of BB in the multimodal treatment of late-stage lymphedema. We did not factor the specific etiology of lymphedema in our patient population, which may affect the overall outcomes. Additionally, patients were selected based on their compliance with follow-up, which is likely to have an influence in our results. Further assessment of our triple therapy surgical treatment algorithm includes the use of validated patient-reported outcome measurement tools to assess for patient satisfaction and quality of life, which is ultimately the primary endpoint to optimize from the patient experience.

## 5. Conclusions

Late stage II and III lymphedema represents a challenge in predicting and maintaining limb volume normalization. We proposed a triple therapy algorithm for later-stage lymphedema that includes debulking and physiologic procedures with the addition of nanofibrillar collagen scaffold technology. Our authors demonstrate success in achieving normalization of limb volumes and maintenance in patients with late stage II and III lymphedema.

## Figures and Tables

**Table 1:** Patient details.

Patient Details	
Number of patients	14
Average Age	62 ± 12.1 years
Lymphedema Stage	
Late stage 2	11
Stage 3	3
Extremity affected	
Upper extremity	8
Lower extremity	6

**Table 2:** Treatment summary.

Treatment Summary	
Order of Treatment	
Large volume liposuction, then physiologic (VLNT/LVA), then BioBridge placement	N = 3
Simultaneous liposuction with physiologic (VLNT/LVA), then BB	N = 11
Liposuction average volume	
Upper extremity	500 ± 168 cc
Lower extremity	1983 ± 1748 cc
Physiologic surgeries performed	
VLNT + LVA VLNT	N = 6 N = 6
LVA (1-3 LVAs performed)	N = 2

**Table 3:** Lymphedema limb volume excess is presented at three time points: baseline, following liposuction and physiologic surgery, and following BB placement. Data are presented in median values with interquartile range. Both percent volume excess (%) and raw volume (cc) are presented.

Limb Volume Excess								
	Baseline		Post Lipo & Physiologic			Post BB Placement		
	%	cc	%	cc	*p*	%	cc	*p*
Total	29 (14, 43)	1086 (585, 1554)	0.5 (−4.3, 3.8)	26 (−187, 130)	0.0001	−1 (−3.3, 1.3)	−36 (−216, 30)	0.0001
UE	34 (17, 44)	629 (406, 1010)	1.0 (−4.3, 9.0)	26 (−111, 126)	0.0078	0.0 (−2.5, 2.0)	−5 (−38, 47)	0.0078
LE	24 (20, 37)	1696 (1128, 3029)	−0.5 (−4.5, 2.3)	−57 (−343, 156)	0.0313	−2.5 (−4.3, −0.5)	−209 (−346, −57)	0.0313
VLNT	26 (16, 34)	870 (339, 1218)	−1.0 (−6.0, 3.8)	−31 (−257, 139)	0.0156	−1.0 (−3.5, 3.5)	−24 (−117, 122)	0.0313
VLNT + LVA	28 (19, 39)	1487 (574, 3029)	−0.5 (−4.3, 4.0)	−57 (−212, 132)	0.0313	−2.5 (−5.5, −0.5)	−209 (−353, −74)	0.0313
